# One-Volt, Solution-Processed Organic Transistors with Self-Assembled Monolayer-Ta_2_O_5_ Gate Dielectrics

**DOI:** 10.3390/ma12162563

**Published:** 2019-08-12

**Authors:** Navid Mohammadian, Sheida Faraji, Srikrishna Sagar, Bikas C. Das, Michael L. Turner, Leszek A. Majewski

**Affiliations:** 1 School of Electrical and Electronic Engineering, University of Manchester, Sackville Street, Manchester M13 9PL, UK; 2 School of Chemistry, University of Manchester, Oxford Road, Manchester M13 9PL, UK; 3 School of Physics, Indian Institute of Science Education and Research, Thiruvananthapuram, Kerala 695551, India

**Keywords:** organic thin-film transistor (OTFT), one-volt operation, tantalum oxide, anodization, self-assembled monolayer (SAM) modification

## Abstract

Low-voltage, solution-processed organic thin-film transistors (OTFTs) have tremendous potential to be key components in low-cost, flexible and large-area electronics. However, for these devices to operate at low voltage, robust and high capacitance gate dielectrics are urgently needed. Herein, the fabrication of OTFTs that operate at 1 V is reported. These devices comprise a solution-processed, self-assembled monolayer (SAM) modified tantalum pentoxide (Ta_2_O_5_) as the gate dielectric. The morphology and dielectric properties of the anodized Ta_2_O_5_ films with and without *n*-octadecyltrichlorosilane (OTS) SAM treatment have been studied. The thickness of the Ta_2_O_5_ film was optimized by varying the anodization voltage. The results show that organic TFTs gated with OTS-modified tantalum pentoxide anodized at 3 V (d ~7 nm) exhibit the best performance. The devices operate at 1 V with a saturation field-effect mobility larger than 0.2 cm^2^ V^−1^ s^−1^, threshold voltage −0.55 V, subthreshold swing 120 mV/dec, and current on/off ratio in excess of 5 × 10^3^. As a result, the demonstrated OTFTs display a promising performance for applications in low-voltage, portable electronics.

## 1. Introduction

Organic thin-film transistors (OTFTs) have been attracting interest owing to their developing applications in low-cost, light-weight, and flexible electronic devices such as active-matrix displays [1], radio frequency identification tags [2], and bio and chemical sensors [3]. Over the years, progress in semiconducting materials and fabrication techniques has dramatically increased the charge mobility in these devices to well above 1 cm^2^ V^−1^ s^−1^ [4,5]. However, in general, these devices operate with relatively high gate voltages (V_G_ ≥ 3 V) which hinders their integration in low-voltage, portable applications [6]. In order to enable organic TFTs that operate below 3 V and deliver high output current, the use of ultra-thin, high dielectric constant (high-κ) materials such as HfO_2_, Ta_2_O_5_, TiO_2_, ZrO_2_, Al_2_O_3_, Y_2_O_3_, CeO_2_ [7,8,9] is required. In particular, tantalum pentoxide (Ta_2_O_5_) is a very promising candidate due to the high dielectric constant in the bulk (κ_bulk_ ~27) and as a thin-film (κ_thin-film_ ~20). These values are at least two times larger than that of Al_2_O_3_ (κ_bulk_ ~9) [10] and five times larger than that of SiO_2_ (κ_bulk_ ~3.9). As a result, Ta_2_O_5_ has been abundantly used in electrolytic capacitors, DRAM devices, and recently in solution-processed inorganic semiconductor thin-film transistors as a promising gate dielectric for low-power electronics [11]. 

A common way to deposit a high-quality, pinhole-free film of tantalum oxide is by atomic layer deposition (ALD). However, ALD is a relatively slow process and requires a high vacuum which is not suitable for low-cost, large-area deposition [12]. Alternatively, magnetron sputtering deposition (MSD) has been used to form tantalum oxide films [13]. However, it is a time-consuming, high vacuum technique which suffers from the same drawbacks as ALD. Recently, anodic oxidation (so-called anodization) has attracted a lot of attention because it is a low-cost, solution-based deposition process that can be performed under ambient conditions [14]. Anodization is a self-limiting and self-healing process that gives pinhole-free, homogenous oxide layers that can be grown in an ambient atmosphere at room temperature. Previously, low voltage poly(3-hexylthiophene-2,5-diyl) (P3HT) TFTs operating at 3 V using thick (d > 100 nm), e-beam deposited tantalum oxide films have been reported [15]. However, the demonstrated p-channel transistors displayed relatively low mobility (~0.02 cm^2^ V^−1^ s^−1^), positive threshold voltage (+0.26 V), large subthreshold swing (> 1 V/dec), and their on/off current ratios were just above 10. This shows that the fabrication of high performance, organic semiconductor TFTs that can be operated at or below 1 V is not trivial.

To operate OTFTs with gate voltages (V_G_) ≤ 1 V, low values of threshold voltage (V_TH_) and subthreshold swing (SS) are essential. Ideally, V_TH_ should be around 0 V and SS close to 60 mV/dec, which is the theoretical limit of subthreshold swing at room temperature [16]. This is a very challenging task, as it requires the gate dielectric thickness of high-κ materials to be reduced below 10 nm. Such thin insulator layers result in very high leakage currents, especially in Ta_2_O_5_ which has a comparatively small band gap (4.5 eV) when compared with other metal oxide dielectrics such as ZrO_2_, (7.8 eV) or Al_2_O_3_ (8.9 eV). In addition, as in the case of other dielectric metal oxides, the surface hydroxyl (–OH) groups on the surface of tantalum oxide tend to create a large number of insulator/semiconductor interfacial traps which consequently imposes detrimental effects on TFT performance [17]. An effective method to passivate thin layers of metal oxide dielectrics in thin-film transistors is by making the surface considerably less polar via salinization. In this process, organofunctional silane molecules react with surface hydroxyl end groups to form a self-assembled monolayer (SAM). *n*-octadecyltrichlorosilane (OTS) is a silane derived SAM that is conventionally used for metal oxide surface modification. It has been shown that OTS significantly improves dielectric/semiconductor interface in organic TFTs that leads to the reduction of charge carrier traps, and in consequence, to higher charge carrier mobility [18]. Although OTS improves the electrical performance of OTFTs, there is a trade-off as the OTS-modified dielectric is thicker, and hence has a smaller capacitance. Therefore, to optimise the performance of organic transistors, different dielectric thicknesses of Ta_2_O_5_ should be considered.

In this paper, we fabricate and study tantalum oxide capacitors to find the optimum thickness of Ta_2_O_5_ for thin-film transistor applications. Then, we fabricate organic TFTs using solution-processed, ultra-thin (~7 nm) OTS-treated Ta_2_O_5_ as the gate dielectric and DPPDTT-PMMA blend as the active layer, respectively. The optimized transistors exhibit highly reproducible characteristics with virtually no hysteresis, a saturation field-effect mobility μ_sat_ = 0.22 cm^2^ V^−1^ s^−1^, threshold voltage V_TH_ = −0.55 V, subthreshold swing SS = 120 mV/dec, I_on_/I_off_ ratio > 5 × 10^3^, and leakage current (I_G_) of approximately 1 nA at 1 V. The fabricated devices show typical p-channel transistor behaviour, demonstrating a high potential to use the developed process for practical organic TFT fabrication.

## 2. Experimental Section

**Figure 1:**
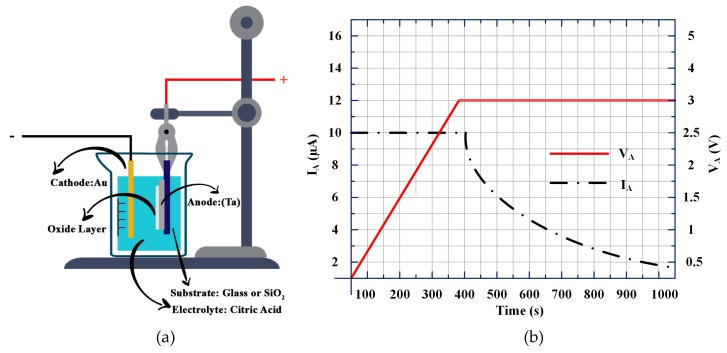
(**a**) Schematic representation of the anodic oxidation process. (**b**) Anodization voltage (V_A_) and current (I_A_) vs. time (t) for the 3 V case study of Ta anodization.

To optimise the dielectric performance of Ta_2_O_5_ and Ta_2_O_5_/OTS films, metal-insulator-metal (MIM) capacitors were fabricated. First, a 100 nm of Ta layer was deposited through a shadow mask by radio frequency (r.f.) magnetron sputtering to serve as the gate electrode. Sputtering was achieved by using a 2-inch diameter Ta target (99.99%) in Ar with a total pressure of 0.5 Pa. Next, samples were anodized in 1 mM of citric acid (≥ 99.5%, Sigma-Aldrich, St. Louis, MO, USA) using 99.99% pure Au wire as the cathode electrode as shown in Figure 1a. To form tantalum oxide films of various thicknesses, anodization voltages (V_A_) of 40, 30, 20, 10, 5 and 3 V with constant current density (0.01 mA/cm^2^) were applied to the anode by Keithley 2400 Source Meter. The process was continued until the ionic current density dropped to 0.005 mA/cm^2^. The variation of anodization voltage and current vs. time is depicted in Figure 1b. Based on the anodization ratio of Ta reported in the literature [19] ([c]_Ta_ = 2.2 nm/V), a 3 V anodized Ta should result in an approximately ~6.6 nm thick Ta_2_O_5_ layer. Some of the as-prepared Ta/Ta_2_O_5_ substrates were treated with O_2_ plasma for 2 minutes and then immersed in a freshly prepared 1 mM solution of OTS in anhydrous toluene at room temperature for 30 minutes. Afterwards, the substrates were removed from the solution and rinsed thoroughly and without interruption with pure toluene in order to get rid of the excess of unreacted OTS. The treated substrates were thereafter annealed on a hot plate at 120 °C for 1 h under a flow of dry N_2_. To obtain MIM capacitors, 50 nm thick Au electrodes were deposited onto the prepared Ta/Ta_2_O_5_ and Ta/Ta_2_O_5_/OTS films. The electrical characterization of the fabricated capacitors was carried out using an Agilent E4980A LCR meter. For the duration of the measurements, 1 V bias was applied to the bottom Ta electrode and the top Au electrode was grounded. Organic TFTs were fabricated in the bottom gate, top contact structure on an ultra-flat, quartz-coated glass substrate (S151, Ossila, Sheffield, UK). Poly(3,6-di(2-thien-5-yl)-2,5-di (2-octyldodecyl)-pyrrolo [3,4-c] pyrrole-1,4-dione) thieno [3,2-b] thiophene) (DPPDTT) and poly(methyl methacrylate) (PMMA) and their blend solutions were prepared according to the procedures reported elsewhere [20]. In short, the DPPDTT-PMMA blend solution was prepared by separately dissolving 0.5 wt% DPPDTT and 0.5 wt% of PMMA in 1,2-dichlorobenzene (DCB) and subsequently mixed in a 7:3 ratio. Afterwards, the blend was stirred until completely mixed. The blend was then spin-coated at 2000 rpm for 2 min on substrates pre-heated at 100 °C. Subsequently, the deposited films were annealed at 100 °C for 30 min to 1 h under N_2_ flow. Finally, a 100 nm thick layer of 99.99% Au was thermally evaporated through shadow masks to serve as a source and drain electrodes.

The channel width (W) and length (L) were 1 mm and 30 μm, respectively. The electrical characterization of the fabricated OTFTs was carried out using an Agilent E5270B semiconductor analyser (Santa Clara, CA, USA) and E4980A LCR meter (Keysight, Santa Rosa, CA, USA) at room temperature. The field-effect mobility is calculated by applying a linear fit to I_D_^1/2^ vs V_G_ in the saturation regime using Equation (1):

(1) $$I_{D}=\left(\frac{C_{i}{W\mu }_{\mathrm{SAT}}}{2L}\right){\left(V_{G}-V_{T}\right)}^{2}$$

where *V_G_* is the gate voltage, *V_T_* is the threshold voltage, *C_i_* is the gate capacitance density, $\mu_{SAT}$ is saturation field-effect mobility, and *W* and *L* are channel width and length, respectively. All electrical measurements were done in ambient conditions in an electrically shielded, dark box.

## 3. Results and Discussions

**Figure 2:**
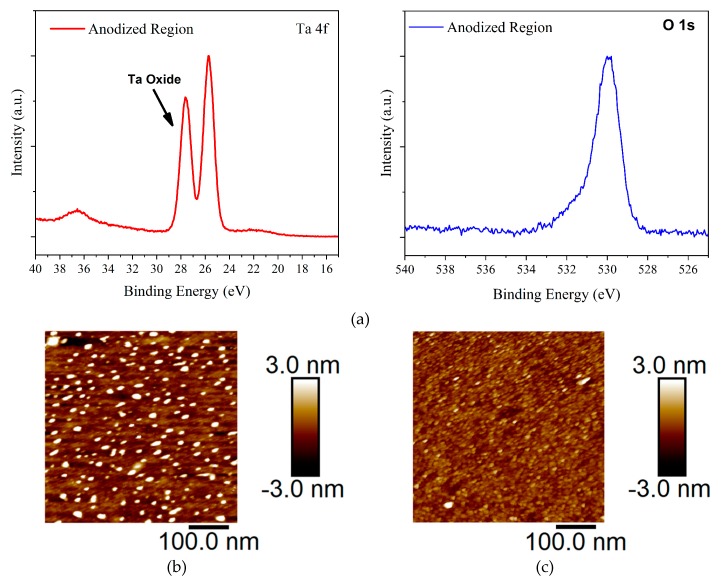
(**a**) Ta 4f and O 1s XPS spectra of the fabricated Ta_2_O_5_ films. (**b**) AFM topography image of untreated Ta_2_O_5_ and (**c**) OTS-treated Ta_2_O_5_ films, respectively.

The presence of anodized tantalum oxide films was confirmed by X-ray photoelectron spectroscopy (XPS, Axis Ultra Hybrid, Kratos Analytical, Manchester, UK). A thick, i.e., thicker than the sampling depth of XPS, oxide was observed on the anodized region of the samples (see Figure 2a and Figures S1–S3 in the Supplementary Materials). By analysing the area of the Ta 4f and O 1s peaks, the Ta and O ratio was found to be nearly 2:5 confirming that tantalum pentoxide (Ta_2_O_5_) was successfully prepared through anodization. Also, as shown in Figure 2a, the normalized O 1s peak fit indicates that the oxide formation on the anodized regions is consistent with the peak fitting analysis of the Ta 4f regions. To investigate the surface morphology of the anodized Ta_2_O_5_, atomic force microscope (AFM, Multimode, Bruker UK, Coventry, UK) images of the fabricated films have been taken. Figure 2b,c show surface topography of both untreated and OTS-treated Ta_2_O_5_ films, respectively. The RMS roughness of untreated Ta_2_O_5_ film is 2.5 nm and the OTS-treated Ta_2_O_5_ 1.3 nm. The tantalum oxide grown by anodization appears to be homogenous, uniform and fairly smooth. However, a more detailed AFM topographical analysis shows that OTS surface modification has made the oxide surface somewhat smoother when compared with as-prepared Ta_2_O_5_ films.

**Figure 3:**
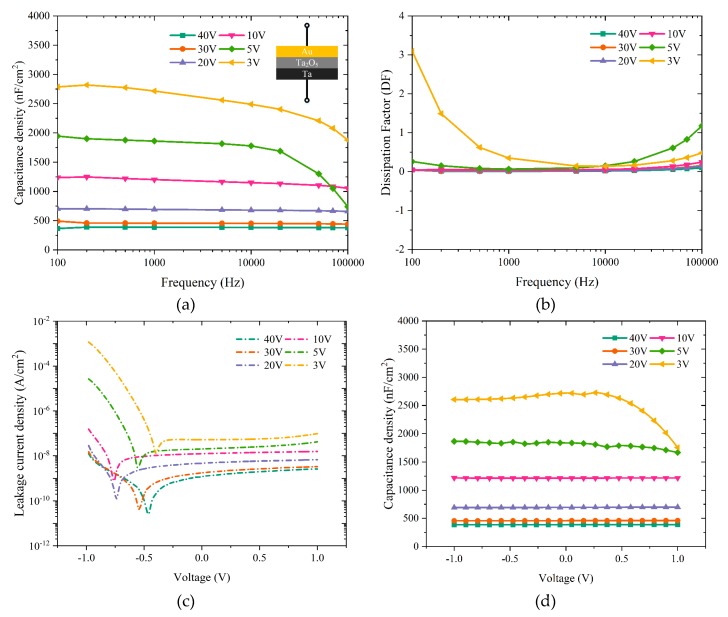
(**a**) Capacitance density and (**b**) dissipation factor vs. frequency, as well as (**c**) leakage current density and (**d**) capacitance density vs. voltage characteristics of the studied Ta/Ta_2_O_5_/Au capacitors.

Figure 3 illustrates capacitance density, dielectric loss, leakage current and capacitance-voltage curves of anodized Ta_2_O_5_ films that were obtained at different anodization voltages, i.e., 40, 30, 20, 10, 5 and 3 V. In order to verify the measured values and test the devices’ fabrication reproducibility, all measurements were carried out on 20 randomly chosen devices out of the 100 different capacitors with Ta/Ta_2_O_5_/Au structure (cf. inset of Figure 3a). Figure 3 shows the response of one typical device out of the 20 studied devices. Figure 3a depicts the capacitance density vs. frequency from 100 Hz to 100 kHz for the corresponding anodization voltages. As can be seen, for the dielectric thicknesses greater than ~22 nm (V_A_ = 10 V), capacitance density is relatively constant across the studied frequency range. As the Ta_2_O_5_ thickness decreases, it appears that the capacitors become unstable at both ends of the chosen frequency spectrum. Capacitors fabricated with Ta_2_O_5_ anodized at 40, 30, and 20 V exhibited an average capacitance density of 380, 450, and 700 nF/cm^2^ at 1 kHz, respectively. The maximum standard deviation of the measured capacitance values for these capacitors was ±20 nF/cm^2^.

On the other hand, the capacitors with anodic Ta_2_O_5_ prepared at 10, 5 and 3 V show much higher capacitances but also significantly higher leakage currents and dielectric losses, especially for V_A_ ≤ 5 V. As a result, we conclude that the pristine Ta_2_O_5_ films anodized at or below 10 V (d ~ 22 nm) are unsuitable for practical applications. Correspondingly, the dissipation factor (DF) measured on capacitors using Ta_2_O_5_ anodized at various voltages is depicted in Figure 3b. For the simplest actual model, i.e., equivalent series resistance in series with the capacitance that neglects equivalent series inductance and insulation resistance, DF is the ratio of equivalent series resistance (ESR) and capacitive reactance (Xc), as written in Equation (2) [21]:

(2) $$\mathrm{DF}=\frac{\mathrm{ESR}}{X_{c}}$$

Ideally, for a given capacitor DF should be zero, but practically it consists of a negligible loss due to resistive characteristics. As shown in Figure 3b, the capacitors with 40, 30, and 20 V anodized Ta_2_O_5_ display a minimal DF that is close to zero. However, as the anodization voltage decreases below 20 V, DF becomes larger and the leakage currents through the anodic dielectric layer start to be appreciable (cf. Figure 3b,c). Accordingly, as the Ta_2_O_5_ dielectric thickness decreases even further (V_A_ = 5 and 3 V) the leakage current through the dielectric becomes very large. In particular, capacitors with the thinnest anodic Ta_2_O_5_, i.e., anodized at 3 V, exhibit the largest leakage current density (J_L_) 10^−3^ A/cm^2^ at 1 V. The same electronic behaviour of the anodized Ta_2_O_5_ can also be seen in the C–V characteristics of the corresponding capacitors shown in Figure 3d, where high leakage currents reduce the voltage across the capacitors resulting in a lower electric field and lower capacitance at the positive end of the C–V curves. This is in contradiction with an ideal case, where a capacitor shows a flat capacitance-voltage characteristic which guarantees reliable and stable device performance. Nevertheless, thicker layers of Ta_2_O_5_ show flat and stable electrical behaviour across the chosen voltage characterization range. Based on the abovementioned dielectric characteristics, it appears that the minimum anodization voltage of tantalum for capacitor and TFT applications is 20 V, which corresponds to approximately 45 nm of Ta_2_O_5_. Indeed, the J_L_ for capacitors with 45 nm Ta_2_O_5_ layers is well below the maximum allowable value for TFT leakage current density (i.e., 10^−6^ A/cm^2^) and is measured to be 10^−7^ A/cm^2^ at 1 V.

**Figure 4:**
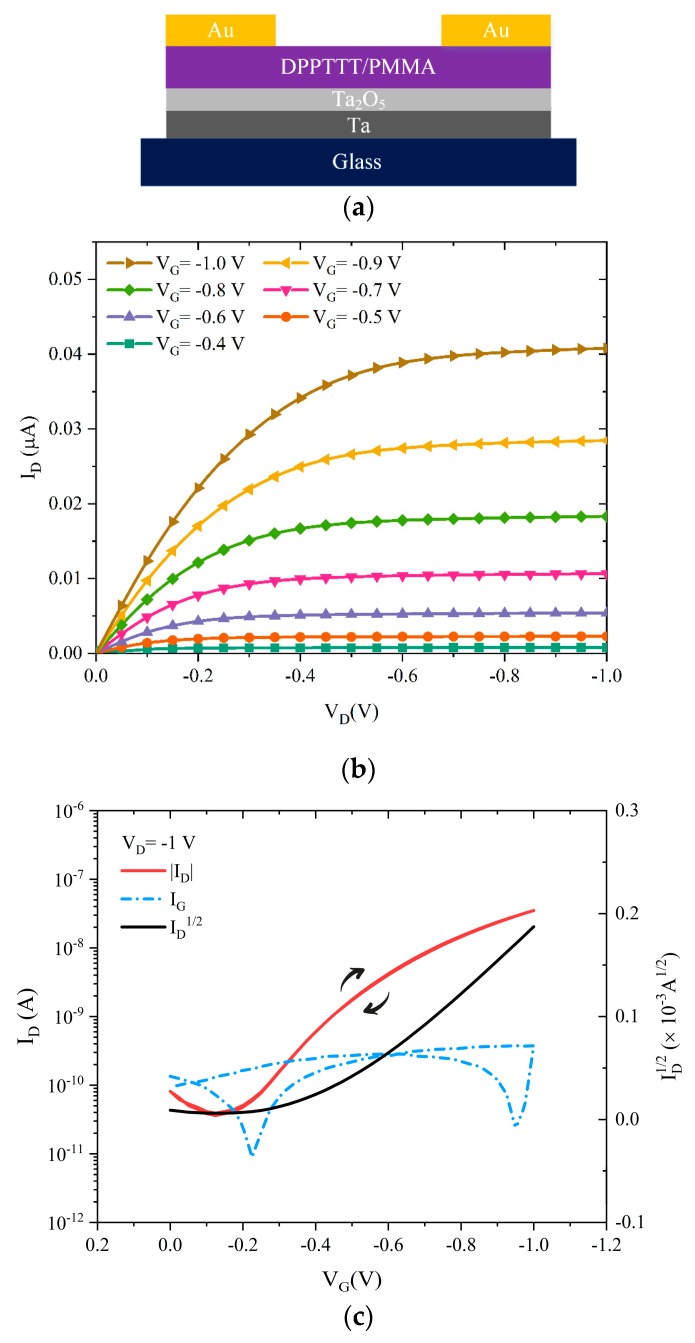
(**a**) Schematic structure of the OTFTs using bare Ta_2_O_5_ anodized at V_A_ = 20 V. (**b**) Output, and (**c**) transfer characteristics of the studied OTFTs including I_D_^1/2^ and I_G_ vs. V_G_ at V_D_ = −1, respectively.

Figure 4a shows the structure of the fabricated bottom-gate, top-contact Ta/(45 nm Ta_2_O_5_)/DPPDTT-PMMA/Au TFTs on a glass substrate (V_A_ = 20 V). Typical output and transfer characteristics of the fabricated transistors are shown in Figure 4b, c, respectively. As can be seen in Figure 4b, the drain current displays clear linear, pinch-off and saturation regions for all applied gate voltages.

The devices operate at 1 V with a saturation field-effect mobility of 0.02 cm^2^ V^−1^ s^−1^, threshold voltage −0.35 V, subthreshold swing 210 mV/dec, and current on/off ratio ~10^3^. This transistor performance is comparable to the previously reported organic TFTs that used SAM-modified, anodized Al_2_O_3_ [22].

To see if the dielectric SAM modification strategy also results in improved transistor characteristics of the TFTs with anodized Ta_2_O_5_, DPPDTT-PMMA TFTs with OTS-modified tantalum pentoxide have been fabricated. OTS is one of the most used SAMs in organic transistors. It has been shown that OTS significantly improves dielectric/semiconductor interface by passivating the gate insulator surface that leads to the reduction of charge carrier traps, and as result, to higher charge carrier mobility [23,24]. During silanization, OTS molecules are attached to the dielectric surface through the chemical reaction of –SiCl with –OH groups on the metal oxide surface. This results in –Si–O–M structures. The other two –SiCl bonds of the OTS molecule react with proximate OTS molecules which forms a cross-linked monolayer. The OTS-treatment of the Ta_2_O_5_ layer inevitably results in a thicker overall dielectric layer, and hence decreases the overall areal capacitance of the dielectric film. The anodized Ta_2_O_5_ layer should be thin enough to provide the minimum required capacitance and leakage to operate the OTFTs at low voltage. Taking into account that the Ta native oxide thickness is about 3.5 nm [25], it is believed that to suppress the effect of the native oxide on the dielectric properties of Ta_2_O_5_ the thickness of the anodized Ta should be at least 3.5 nm thick. As shown in Figure 3a,c, an untreated Ta_2_O_5_ layer anodized at 3 V (d ~7 nm) exhibits a large enough areal capacitance to be able to operate DPPDTT-PMMA TFTs at 1 V. However, the very high leakage current density of these thin layers would lead to poor field-effect transistor characteristics if used as prepared.

**Figure 5:**
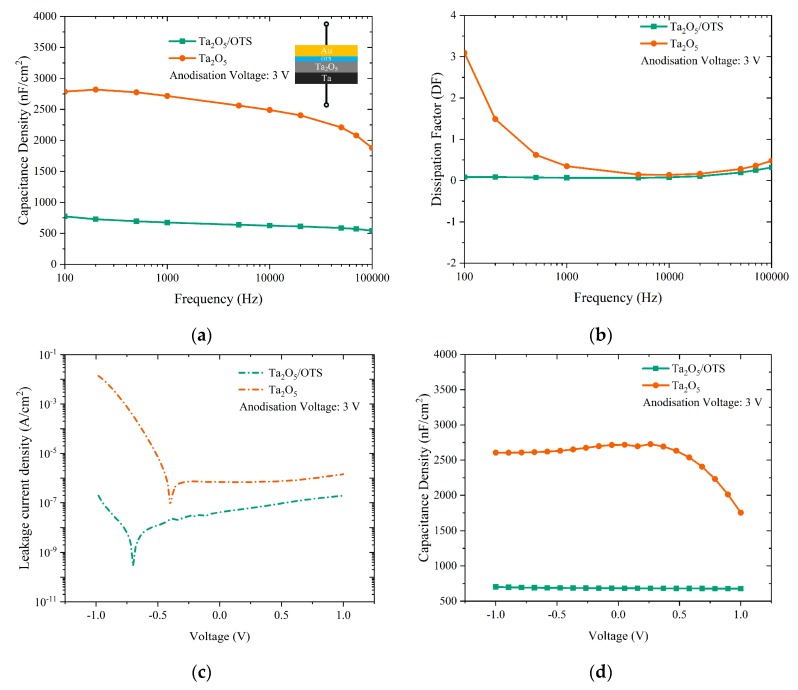
(**a**) Capacitance density and (**b**) dissipation factor vs. frequency, as well as (**c**) leakage current density and (**d**) capacitance density vs. voltage characteristics of the 3 V anodized Ta/Ta_2_O_5_/Au capacitors with and without OTS treatment.

Figure 5a–d compare the dielectric properties of 3 V anodized untreated Ta_2_O_5_ and OTS-treated Ta_2_O_5_ films. As can be observed in Figure 5a, the capacitance density of OTS-treated Ta_2_O_5_ is notably reduced from 2700 nF/cm^2^ for bare Ta_2_O_5_ film to approximately 680 nF/cm^2^. This is due to the addition of the OTS layer which leads to a thicker dielectric film and correspondingly smaller overall capacitance density because of two dielectric layers in series. Nonetheless, capacitance density curves for OTS-treated Ta_2_O_5_ are highly stable over a wide range of frequencies. The dielectric loss (Figure 5b) for OTS-treated Ta_2_O_5_ shows negligible variation (less than 0.1) confirming a low defect density in the OTS-treated Ta_2_O_5_ films. As shown in Figure 5c, leakage current density for OTS-treated Ta_2_O_5_ is approximately 2 × 10^−7^A/cm^2^ at ±1 V; this is reduced by circa five orders of magnitude at −1 V and circa one order of magnitude at +1 V when compared to films of untreated Ta_2_O_5_ of comparable thickness. The leakage current of the OTS-treated Ta_2_O_5_ is somewhat asymmetric and slightly lower for negative voltages. This is very likely caused by the use of electrodes with different work functions and differences in the roughness of the Ta/Ta_2_O_5_ and Ta_2_O_5_/OTS interfaces [26]. In addition, as illustrated in Figure 5d, in contrast to untreated Ta_2_O_5_, OTS surface modification has improved the stability of the capacitance density over the studied voltage range (−1 V to 1 V) and led to almost ideally flat C–V curves.

**Figure 6:**
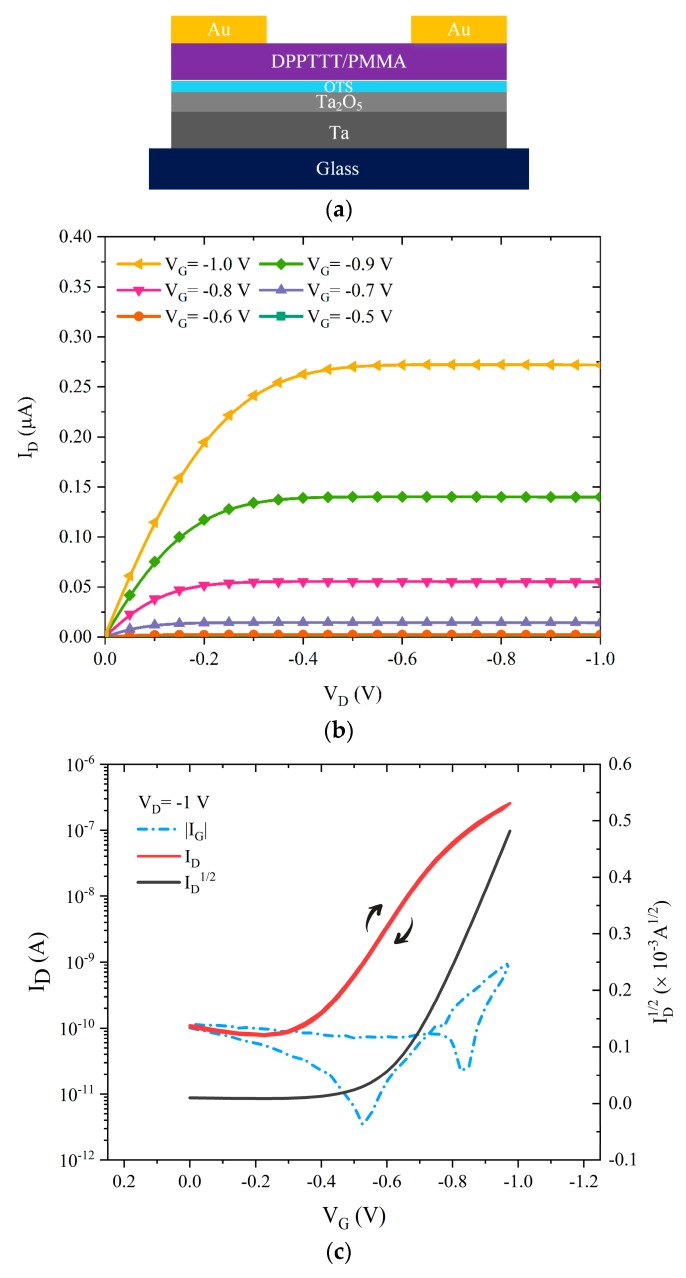
(**a**) Schematic structure of the OTFTs using OTS-modified Ta_2_O_5_ anodized at V_A_ = 3 V. (**b**) Output and (**c**) transfer characteristics of the fabricated OTFTs including I_D_, I_D_^1/2^ and I_G_ vs. V_G_ at V_D_ = −1 V, respectively.

To test if the optimized, OTS-modified Ta_2_O_5_ layers result in well-working 1 V devices, glass/Ta/(7 nm Ta_2_O_5_)/OTS/DPPDTT-PMMA/Au OTFTs have been fabricated (V_A_ = 3 V). Typical output and transfer characteristics of the fabricated transistors are shown in Figure 6b,c, respectively. The optimized devices operate at 1 V with virtually no hysteresis, the saturation field-effect mobility is 0.22 cm^2^ V^−1^ s^−1^, the threshold voltage −0.55 V, subthreshold swing 120 mV/dec, and the current on/off ratio is in excess of 5 × 10^3^. Additionally, the leakage current at −1 V is measured to be less than 1 nA at all applied biases. The calculated field-effect mobility for these devices is > 0.2 cm^2^ V^−1^ s^−1^, ten times larger than the analogous DPPDTT-PMMA TFTs fabricated on bare Ta_2_O_5_. It is believed that charge carrier mobility in TFTs is directly affected by the interfacial trap density [27]. Trap density in thin-film transistors can be calculated using Equation (3):

(3) $$N_{it}=\left(\frac{SS\log\left(e\right)}{kT/q}\right)\frac{C_{G}}{q}$$

where *C_G_* is the gate capacitance density, *q* is the electron charge, *k* is the Boltzmann’s constant, *T* is the temperature, and *SS* is subthreshold swing. *N_it_* is calculated to be 1.09 × 10^13^ cm^−2^ and 4.2 × 10^12^ cm^−2^ for OTFTs using 20 V, untreated Ta_2_O_5_ and 3 V, OTS-treated Ta_2_O_5_, respectively. The reduction of trap density confirms that the OTS layers shield the active layer from traps at the anodic Ta_2_O_5_/DPPDTT-PMMA interface. Table 1 compares the key figures-of-merit of the 1 V OTFTs fabricated in this work in comparison with one-volt organic TFTs we reported previously [22,28,29]. As can be seen, the gate capacitance density of the developed OTS-treated Ta_2_O_5_ dielectric layer used here is significantly higher. Importantly, other transistor parameters are comparable or indeed better than the parameters of the state-of-the-art organic TFTs reported to date [30,31,32]. As a result, the demonstrated organic transistors are promising candidates for use in low-voltage, portable electronics.

## 4. Conclusions

In conclusion, solution-processed, low threshold voltage organic thin-film transistors that can be operated at 1 V have been demonstrated. The low operational voltage was achieved by using extremely thin (7 nm), anodized Ta_2_O_5_ films that were modified by the application of an OTS SAM. The optimized DPPDTT-PMMA TFTs display threshold voltages around −0.55 V, low subthreshold slopes 120 mV/dec, operate with negligible hysteresis and possess average saturated field-effect mobility in excess of 0.2 cm^2^ V^−1^ s^−1^ at 1 V. This approach has a high potential to enable the design of stable, ultra-low voltage organic semiconductor circuitry in a highly reproducible manner.

## Figures and Tables

**Table 1:** Comparison of the DPPDTT-PMMA OTFTs’ performance with their counterparts in the literature.

Parameters	20 V Untreated	3 V OTS-Treated	[22]	[28]	[29]
Dielectric	Ta_2_O_5_	Ta_2_O_5_	ODTS/Al_2_O_3_	BST-P	BST-CEC/PVP
C_G_ (nF/cm^2^)	700	670	550	94	40
Mobility (cm2 V^−1^ s^−1^)	0.02	0.22	0.1	0.14	0.3
V_TH_ (V)	−0.35	−0.55	−0.45	−0.5	−0.7
SS (V/dec)	220	120	160	221	140
ON/OFF Ratio	10^3^	5 × 10^3^	10^3^	10^3^	10^3^
Operating voltage (V)	−1	−1	−1	−1	−1.5

## Supplementary Materials

The following are available online at https://www.mdpi.com/1996-1944/12/16/2563/s1, Figure S1: Normalized peak fitted Ta 4d spectra for both control and anodized regions of the sample showing only Ta oxide in the anodized region relative to a metal/oxide mixture in the control region. Figure S2: Normalized O 1s spectra for both control and anodized regions. Figure S3: Normalized Ta 4f spectra for both control and anodized regions.
