# Supplementary material for: One-Volt, Solution-Processed Organic Transistors with Self-Assembled Monolayer-Ta2O5 Gate Dielectrics
Source: Materials (Basel). 2019 Aug 12;12(16):2563. doi: 10.3390/ma12162563 (PMC6720892; doi:10.3390/ma12162563)
Supplement: Supplementary file 1 [file materials-12-02563-s001.pdf]

materials

Supplementary Materials

One-Volt, Solution-Processed Organic Transistors with Self-Assembled Monolayer-Ta_2O_5 Gate Dielectrics

Navid Mohammadian [1], Sheida Faraji [2], Srikrishna Sagar [3], Bikas C. Das [3], Michael L. Turner [2] and Leszek A. Majewski [1]

[1] School of Electrical and Electronic Engineering, University of Manchester, Sackville Street, Manchester, M13 9PL, UK
[2] School of Chemistry, University of Manchester, Oxford Road, Manchester, M13 9PL, UK
[3] School of Physics, Indian Institute of Science Education and Research, Thiruvananthapuram, Kerala 695551, India
* Correspondence: leszek.majewski@manchester.ac.uk

XPS analysis of anodized Ta films

In order to confirm that Ta films were successfully oxidized XPS character of the studied samples was carried out. Initial scanning indicated a large build-up of charge on the surface of the samples. To neutralize the charge on the surface a flood gun was used throughout XPS analysis (2 eV at 20 μA). To excite the photoelectrons XPS analysis was carried out using a monochromatic Al K*α* source (1486.6 eV). Typically the Ta 4f line is chosen for analysis; however, given the overlap of this feature with the O 2s line, it was decided to also acquire the Ta 4d spectral line in order to confirm any differences between the analysis regions. A large difference in counts was noted between both spots analyzed which makes quantitative analysis difficult between the two analysis areas.

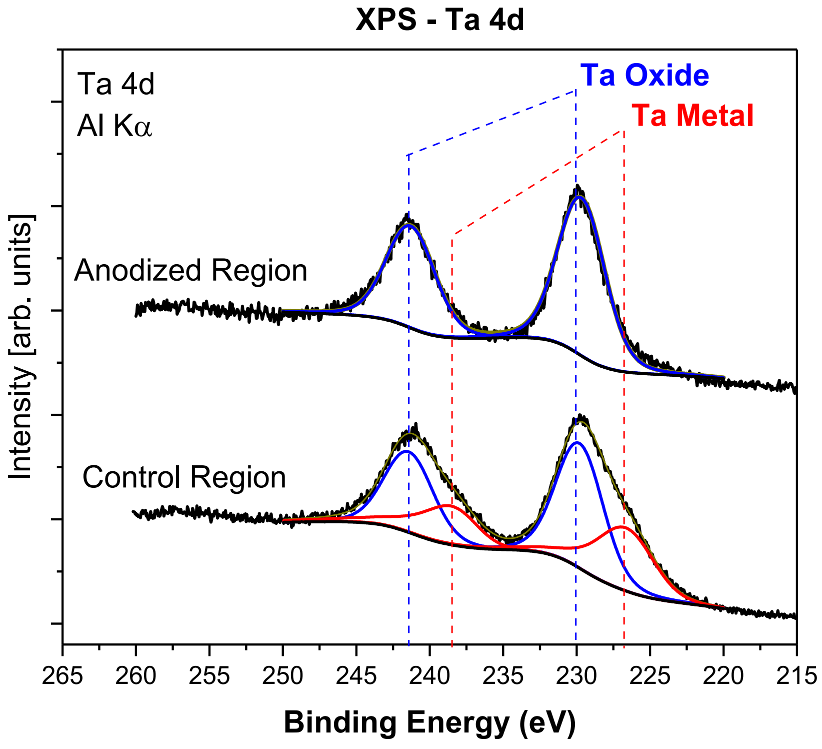

Figure S1. Normalized peak fitted Ta 4d spectra for both control and anodized regions of the sample showing only Ta oxide in the anodized region relative to a metal/oxide mixture in the control region.

Figure S1**Error! Reference source not found.** shows a peak fitted comparison between both regions of the sample for the Ta 4d spectral feature. As can be seen, only Ta oxide appears in the anodized region relative to a mixture of metal and oxide in the control region. Oxide in the control region is most likely a native oxide on the Ta surface due to atmospheric exposure.

Normalized O 1s peak fits, as shown in Figure S2, indicate that the native oxide and oxide formation on the anodized region is similar in chemical make-up, consistent with peak fitting analysis of the Ta 4d regions.

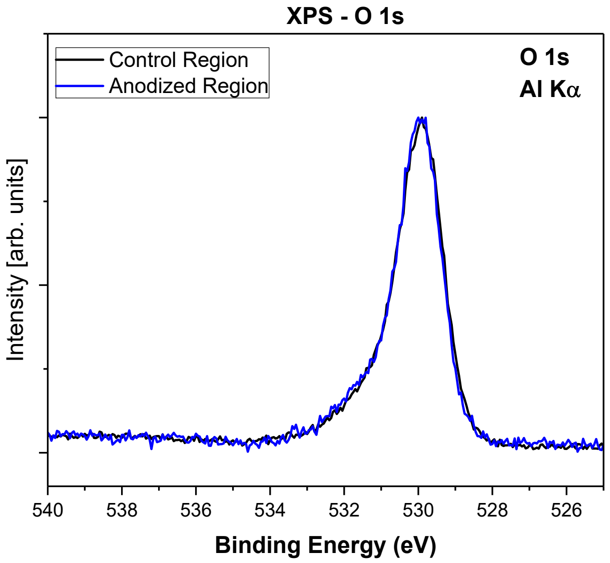

Figure S2. Normalized O 1s spectra for both control and anodized regions.

Additionally, the Ta 4f spectra (which overlap with the O 2s core level) show considerable differences between control and anodized regions that are visible as a pure Ta oxide on the anodized region and a mix of Ta metal and Ta oxide on the control region, as seen in Figure S3.

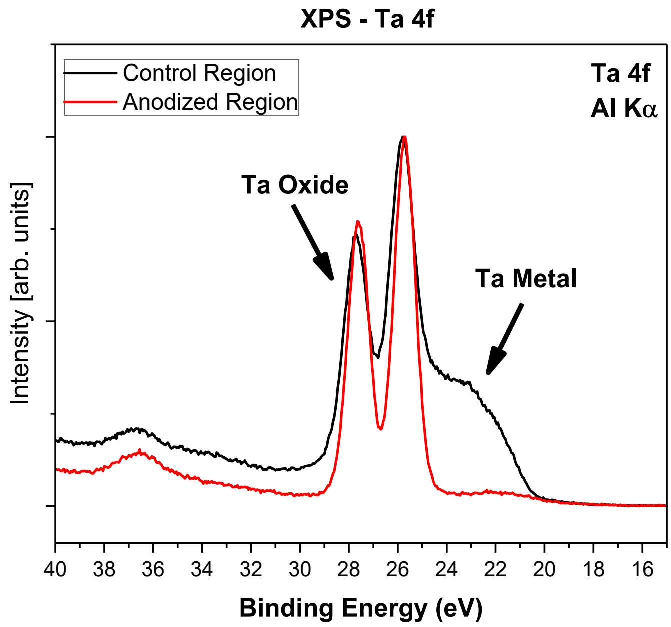

Figure S3. Normalized Ta 4f spectra for both control and anodized regions.

A thick (thicker than the sampling depth of XPS, ~ 5 nm) oxide was observed on the anodized region. This is in contrast to the control region which shows only a thin oxide layer in addition to metallic Ta indicating a native oxide only in this region, most likely due to atmospheric exposure of the Ta metal line.

© 2019 by the authors. Submitted for possible open access publication under the terms and conditions of the Creative Commons Attribution (CC BY) license (http://creativecommons.org/licenses/by/4.0/).
